# Erdheim-Chester Disease Masquerading as Leukemia: Outwitting the Need for Transplant

**DOI:** 10.7759/cureus.94636

**Published:** 2025-10-15

**Authors:** William A Ketterer, Joshua Scott, Zachary Gillooly, Matthew J Rendo

**Affiliations:** 1 Internal Medicine, Wright State University, Dayton, USA; 2 Rheumatology, Wright-Patterson Medical Center, Dayton, USA; 3 Dermatology, Kettering Health Network, Dayton, USA; 4 Hematology/Oncology, Wright-Patterson Medical Center, Dayton, USA; 5 Hematology/Oncology, Brooke Army Medical Center, San Antonio, USA

**Keywords:** braf negative, chronic myelomonocytic leukemia (cmml), cobimetinib, erdheim-chester disease (ecd), fibrinous pleuritis, histiocytosis, kras mutation, monocytosis, neutropenia, xanthelasma

## Abstract

Erdheim-Chester disease (ECD), a rare non-Langerhans cell histiocytosis, involves hyperproliferative histiocytes infiltrating multiple organs. Presentation is highly dependent on the organ system affected, often involving the long bones, central nervous system (CNS), skin, kidneys, and vasculature. Diagnosis is challenging due to diverse manifestations. We present a 54-year-old male with years of discordant symptoms, including psoriasiform lesions, xanthelasmas, recurrent abscesses, arthralgias, and pleural effusions, eventually presenting with diffuse bone pain. Initial evaluation revealed mild lymphocytopenia and monocytosis, prompting a bone marrow biopsy showing a KRAS mutation, raising concerns for chronic myelomonocytic leukemia (CMML). However, a skin biopsy revealed foamy histiocytic infiltration in a xanthelasma. The patient's constellation of symptoms, including skin manifestations, xanthelasmas, microabscesses, pleural effusions, and bone pain, in the context of potential malignancy, cytopenias, monocytosis, and KRAS mutation, suggested ECD. As a rare neoplasm with variable organ involvement, ECD poses a diagnostic challenge. This patient's atypical presentation included diffuse axial skeleton uptake on positron emission tomography (PET), bone pain, absence of bone marrow malignancy, fibrinous pleuritis, xanthelasmas with foamy histiocytes, and a KRAS mutation. While BRAF mutations are common in ECD, RAS isoforms occur in a smaller subset. This case highlights RAS-positive ECD, managed with KRAS pathway-targeted MEK inhibition, in line with the 2019 ECD Medical Symposium recommendations.

## Introduction

As a rare histiocytic disorder with unpredictable clinical manifestations presenting on a spectrum of indolent to life-threatening, Erdheim-Chester disease (ECD) represents a significant diagnostic challenge. Since its initial discovery in 1930, approximately 1,500 cases have been recognized [1]. The prevalence is unknown, but there is a predilection toward males in their fourth to sixth decade of life [2]. Within the past decade, there has been a surge of diagnoses, owing largely to increased awareness of this remarkably rare condition. Diagnosis of ECD is dependent on the identification of distinct histopathologic findings. Classically, tissue lesions demonstrate foamy, lipid-laden histiocytes, commingling fibrosis, and often include Touton giant cells. Radiographically, ECD frequently presents with symmetric osteosclerosis of the lower extremity diaphysis, often referred to as a “hot-knee pattern" [1,3]. The pathophysiology is driven primarily by somatic mutations. These mutations lead to clonal proliferation of histiocytes and an infiltrative, inflammatory process [1-4]. Diagnosis typically involves a multidisciplinary approach as the presentation is quite vague and protean due to the wide array of manifestations dependent on organ involvement, but most commonly with long bone involvement, retroperitoneal fibrosis, cardiovascular, and central nervous system (CNS) infiltration being characteristic and prognostically significant features [1,3].

Due to its proliferative nature, treatments generally entail cancer-targeting therapies. Since 2010, multiple recurrent mutations or rearrangements have been noted to be associated with ECD within the Ras-Raf-MEK-ERK pathway [4-7]. Therapy is guided by the presence of such actionable mutations and disease severity, but targeted therapies are generally reserved for severe, life-threatening, or refractory disease due to potential toxicities and unknown long-term effects. Otherwise, interferon-based regimens remain a mainstay for less aggressive disease [1-5,7,8].

ECD can co-occur with other myeloid malignancies, leading to difficult-to-discern presentations sharing features of both diseases, ranging from unexplained cytopenias or cytosis to bone marrow findings consistent with both histiocytosis and myeloid neoplasia. Both ECD and chronic myelomonocytic leukemia (CMML) frequently harbor mutations in genes involved in clonal hematopoiesis. Given such overlap, a bone marrow biopsy is recommended in ECD patients with any cytopenias or cytosis to rule out concomitant myeloid neoplasms [1,2,3,5,8,9].

This article was previously presented as an abstract at the 2024 Tri-Service Annual Scientific Meeting on September 22, 2024, and the 2025 ACP Internal Medicine Annual Scientific Meeting on April 4, 2025.

## Case presentation

This case report is presented to add to the growing body of literature on the presence of mutations or rearrangements within the Ras-Raf-MEK-ERK pathway, specifically as an example of BRAF-negative, KRAS-positive ECD. The patient is a 54-year-old male with a plethora of presumably unrelated symptoms that, upon further investigation, are likely unified under the diagnosis of ECD. For years, he exhibited an array of symptoms, including the presence of xanthelasmas, rashes with Langerhans cell microabscesses, recurrent fibrinous pleuritis, arthralgias, and progressive neutropenia with monocytosis that, on further reflection, raised appropriate concern for a unifying histiocytic etiology.

**Figure 1:**
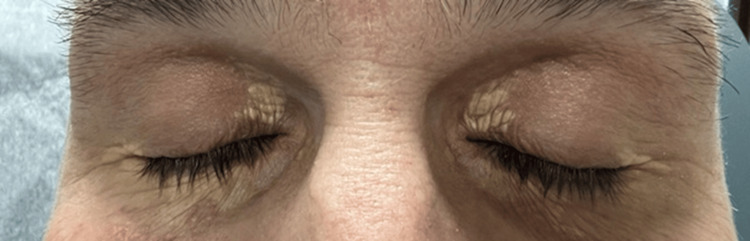
Fitz III with an expected degree of dermatoheliosis for the patient's age. Notable yellowish papules/plaques present on the bilateral upper and lower eyelids, with medial predominance.

In his 40s, the patient began experiencing recurrent, plaque-like erythematous eruptions over his neck and upper back, as well as recurrent microabscesses. Evaluation by dermatology yielded a diagnosis consistent with psoriasis, and the patient would go on to have periodically recurring psoriasiform rashes that improved with topical steroids. Following years of treatment for his recurrent rashes, it was noted that he had developed xanthelasmas around both eyes in the absence of hypercholesterolemia (Figure 1). Concurrently, the patient had been experiencing significant polyarthralgia for a similar time frame as his presentation with psoriasiform lesions. This provoked an evaluation by rheumatology with a subsequent negative workup for underlying rheumatologic disorder (Table 1). Symptoms evolved progressively within the last two years preceding his eventual diagnosis with ECD; sequentially involving recurrent pleuritis with bland pleural effusions, followed by progressive bone pains, cytopenias, and monocytosis. 

**Table 1:** Rheumatologic labs

Lab	Result	Standard reference range (negative/normal)
Rheumatoid factor	16 IU/mL (low titer)	<25 IU/mL
Anti-CCP	Negative	<20 U/mL
14.3.3 eta	Negative	<0.19 ng/mL
Anti-Ro/SSA	Negative	<10 U/mL
Anti-La/SSB	Negative	<10 U/mL

A year preceding his diagnosis, he experienced multiple episodes of left-sided chest pain and dyspnea before being diagnosed with fibrinous pleuritis complicated by recurrent pleural effusions. This eventually led to a video-assisted thoracic surgery (VATS) pleurectomy. Pathology demonstrated fibrinous pleuritis with reactive mesothelial hyperplasia, chronic inflammation, and mild fibrosis. This, along with his prior pleural effusion cytology, was negative for malignancy.

In the months following his VATS pleurectomy, he was found to have a gradually worsening neutropenia with persistent monocytosis and thrombocytopenia (Table 2). These persistent abnormalities prompted a bone marrow biopsy that demonstrated appropriate cellularity, normal hematopoiesis, no dysplasia, but it was notable for a KRAS mutation. At this point, the patient met clinical criteria for CMML with persistent monocytosis (>500/µL), persistent cytopenias, less than 20% blasts on marrow, and evidence of circulating clonality (KRAS mutation) [8]. This resulted in the presumptive diagnosis of CMML and consideration for allogeneic bone marrow transplantation.

**Table 2:** Initial laboratory results demonstrating neutropenia, monocytosis, and thrombocytopenia

Lab	Result	Normal low	Normal high
Absolute neutrophil count	957	1.8 × 10⁹/L	7.7 × 10⁹/L
Absolute monocyte count	1334	0.00 × 10⁹/L	0.80 × 10⁹/L
Platelets	114	150 × 10³/μL	400 × 10³/μL

**Figure 2:**
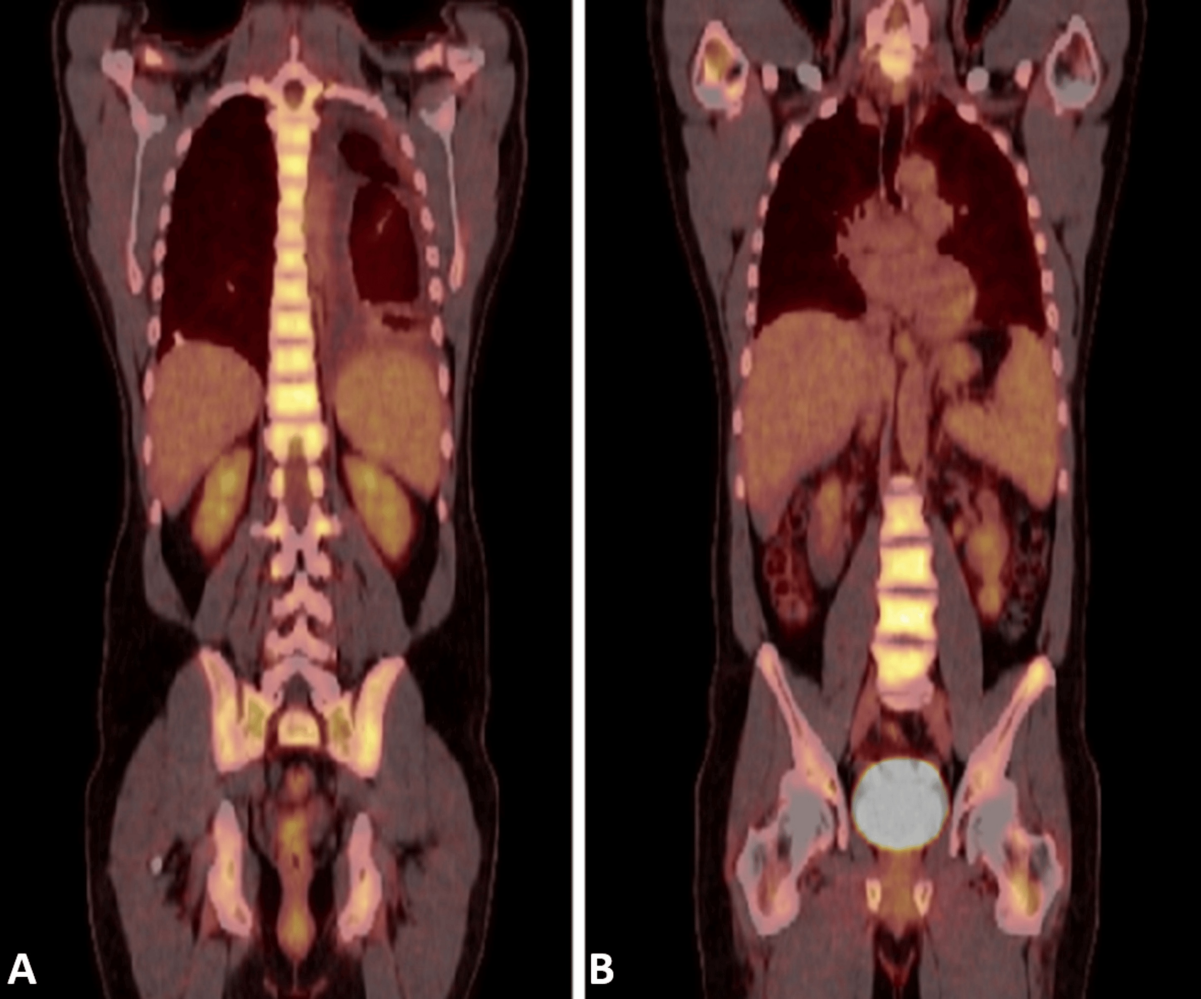
A) Generalized hypermetabolism of the axial skeleton. B) Multifocal hypermetabolic foci throughout the appendicular skeleton.

Seemingly progressive, diffuse bony pains and arthralgias were also prevalent symptoms for the patient throughout this period. This was in the absence of deforming changes or focal etiologies that could potentially explain the transient and unpredictable nature of his pains. PET was obtained and notable for diffuse uptake throughout the axial skeleton (Figure 2). While this was not the typical “hot-knee pattern,” the overwhelming majority of ECD patients show some form of skeletal involvement.

**Figure 3:**
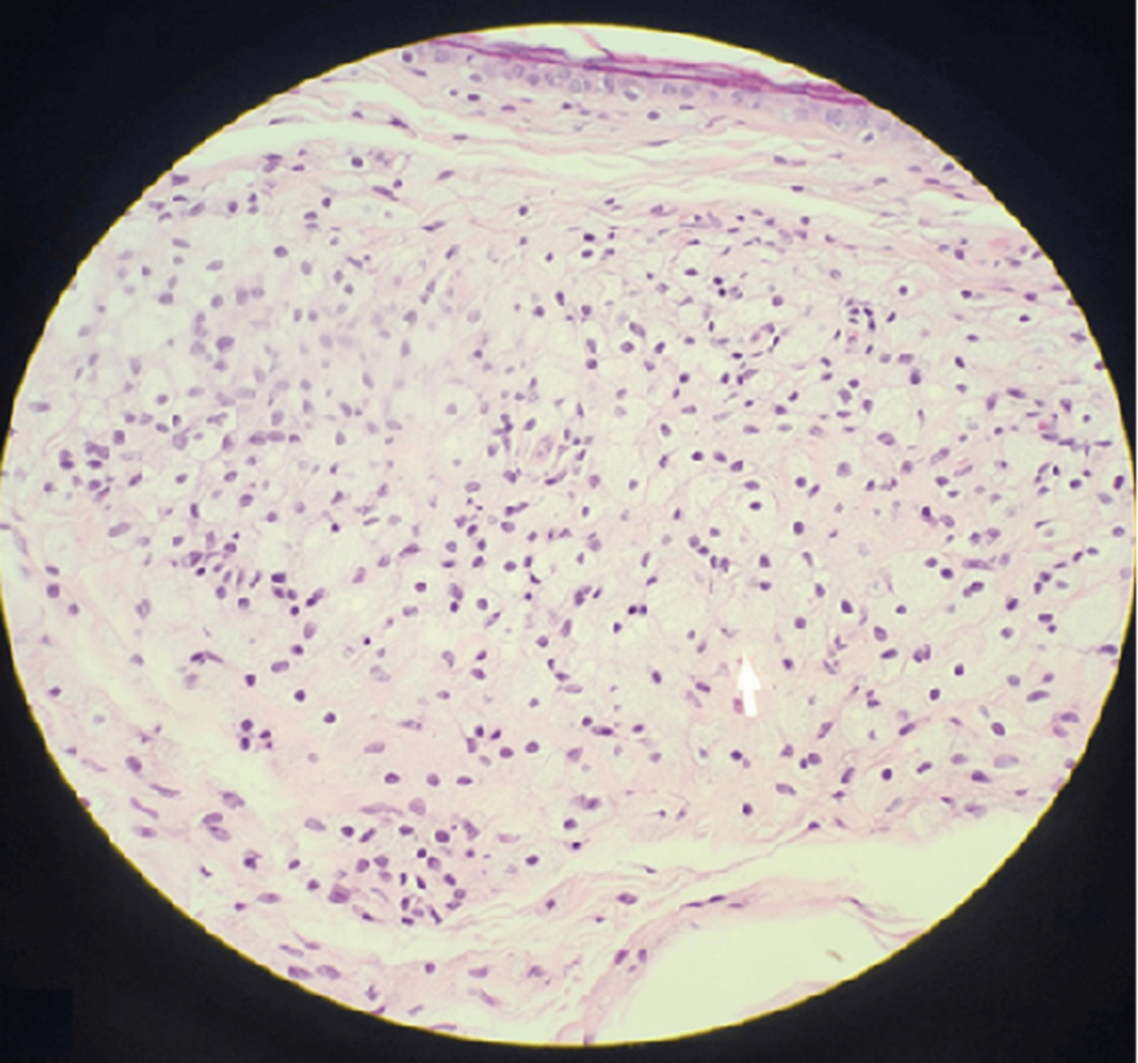
Biopsy findings of xanthelasma pictured in Figure 1 notable for foamy, histiocytic invasion staining CD68 positive and S100 negative.

A biopsy was obtained of his xanthelasma, which was notable for foamy histiocytic invasion staining CD68 positive and S100 negative (Figure 3). A repeat biopsy was obtained at an outside medical center and was indistinguishable from ECD. It was only upon further review of his extensive history of seemingly unrelated symptoms, including multiple skin manifestations initially concerning for psoriasis, xanthelasmas in the absence of hypercholesterolemia, recurrent microabscesses notable for the presence of foamy histiocytes, recurrent pleural effusions resulting in fibrinous pleuritis with eventual pleurectomy, and chronic progressive joint and bone pains, that prompted the suspicion for a unifying process. This constellation of symptoms in the setting of concern for underlying malignancy, cytopenias with monocytosis, and a known KRAS mutation prompted concerns for ECD, and treatment was initiated with cobimetinib. The first cycle of treatment consisted of cobimetinib 60 mg orally once daily for the first 21 days of each 28-day cycle. Initial treatment was well tolerated, with only mild, self-limited diarrhea as the notable adverse effect. Following the first cycle, the patient experienced resolution of monocytosis and fevers, as well as improvement of his cytopenias, myalgias, and arthralgias. 

## Discussion

ECD is a rare, non-Langerhans cell histiocytosis characterized by infiltration of foamy histiocytes in multiple organs, leading to a diverse array of clinical manifestations and often posing a significant diagnostic challenge [1,2,9]. The protean nature of ECD, as highlighted by this case, can result in misdiagnosis and delayed treatment, underscoring the importance of a high index of suspicion and a thorough evaluation of seemingly disparate symptoms. While the classic presentation involves symmetric long bone pain, diabetes insipidus, and neurological symptoms [9,10,11], atypical presentations are increasingly recognized, expanding the clinical spectrum of this disease.

Our patient presented with a complex constellation of symptoms, including diffuse uptake in the axial skeleton on PET scan, associated bone pain, absence of malignancy on bone marrow biopsy, fibrinous pleuritis, xanthelasmas with biopsy demonstrating foamy histiocytic infiltrates (CD68+/S100-), and a circulating KRAS mutation. This presentation deviates from the more commonly reported "hot-knee pattern" and emphasizes the importance of considering ECD even in the absence of typical skeletal findings. While skeletal involvement is present in the majority of ECD patients, the distribution can vary significantly, ranging from diaphyseal sclerosis to diffuse bone marrow infiltration [1,11,12]. The observed axial skeletal uptake in our patient is consistent with reports of atypical skeletal manifestations in ECD, further highlighting the diagnostic challenges [1,11,12].

The presence of xanthelasmas in the absence of hyperlipidemia further contributed to the diagnostic complexity. Xanthelasmas are commonly associated with hypercholesterolemia, but they can also be a feature of histiocytic disorders, including ECD [13-16]. The histopathologic confirmation of foamy histiocytes within the xanthelasma lesions, staining positive for CD68 and negative for S100, was a crucial diagnostic clue, strongly suggesting a non-Langerhans cell histiocytosis [13-16]. This finding prompted further investigation and ultimately led to the correct diagnosis.

The concurrent presence of recurrent fibrinous pleuritis also adds to the uniqueness of this case. Pleural involvement in ECD is not commonly reported, with only a limited number of cases describing pleural effusions or thickening [17,18]. This case is unique as it presented as fibrinous pleuritis, which has rarely been reported as a manifestation of ECD. The underlying mechanisms for pleural involvement in ECD remain unclear but are thought to involve histiocytic infiltration and inflammation of the pleura [17,18]. The absence of malignancy on pleural fluid cytology and VATS pleurectomy further supported the diagnosis of ECD, differentiating it from other potential causes of pleural disease.

**Figure 4:**
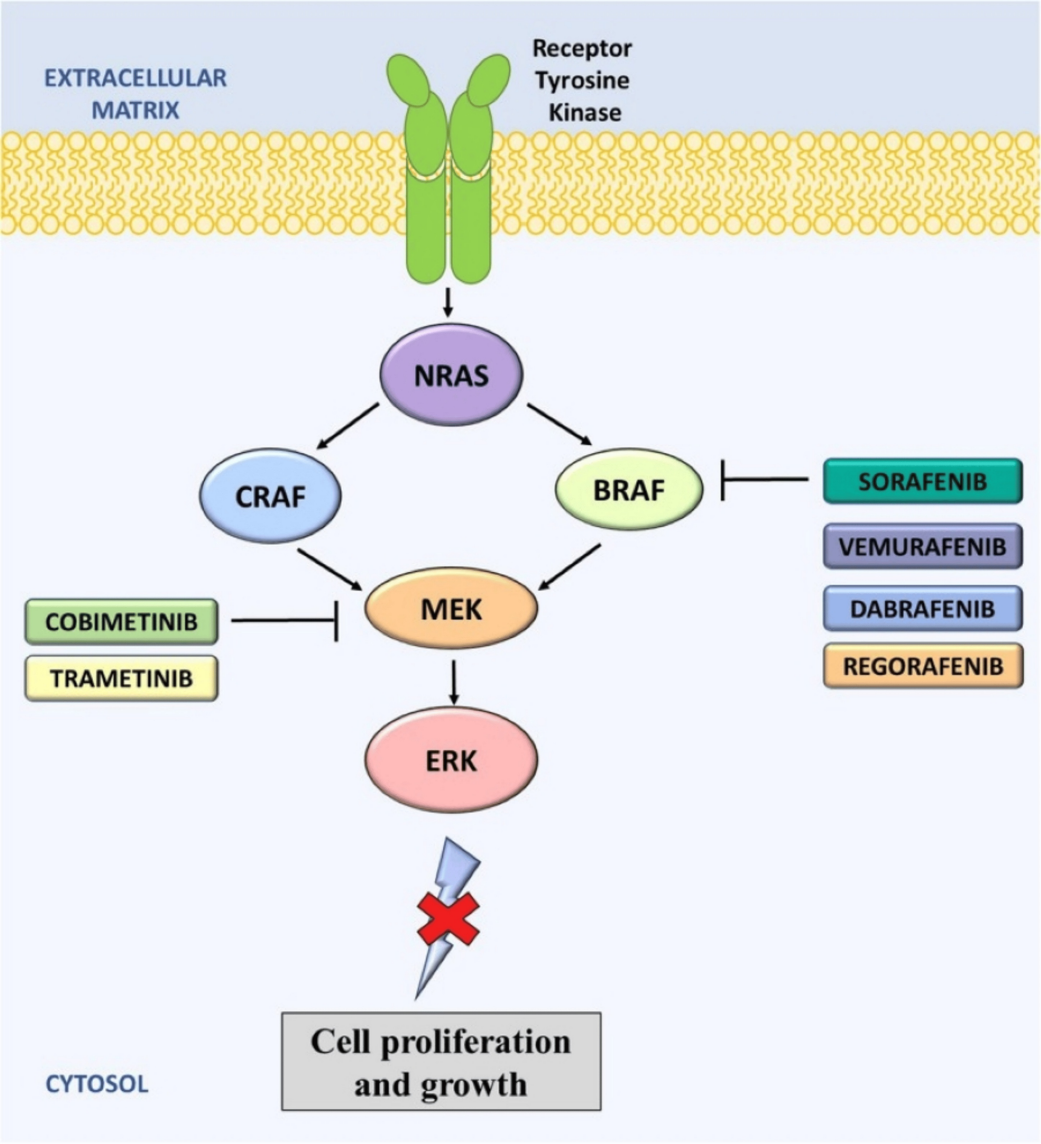
Anti-BRAF drugs, along with MEK inhibitors, target the RAS signaling pathway to inhibit cancer cell proliferation and growth. RAS activates both CRAF and BRAF, and these inhibitors work to block downstream signaling. Image Credit: WikiMedia Commons [7]; licensed under the Creative Commons CC BY-SA 3.0 Attribution-Share Alike 3.0 Unported license (https://creativecommons.org/licenses/by-sa/3.0/deed.en)

The detection of a KRAS mutation in the bone marrow was another significant finding. While BRAF V600E mutations are the most frequently identified driver mutations in ECD, occurring in approximately 50-60% of cases, mutations in the RAS pathway, including KRAS, NRAS, and HRAS, are found in a smaller subset of patients (approximately 5-8%) [4,5,6]. Several studies have demonstrated the presence of RAS mutations in BRAF-negative ECD cases, suggesting an alternative oncogenic mechanism driving disease pathogenesis [5,6,7,19,20]. Our case further supports this notion and emphasizes the importance of comprehensive molecular profiling in ECD patients, particularly those without BRAF mutations, to identify potentially targetable RAS mutations. The identification of KRAS mutation ultimately helped direct the treatment course, although clinical trials using KRAS direct inhibitors have demonstrated little efficacy in treating KRAS-mutated cancers [6,19,20]. As such, the MEK inhibitor cobimetinib was used as the mutation was further upstream within the Ras-Raf-MEK-ERK pathway (Figure 4) and has demonstrated promise for treating ECD [6,7,19,20].

The fact that this patient was initially diagnosed with CMML highlights the significant diagnostic challenges posed by ECD. More importantly, this patient was set upon the pathway for bone marrow transplantation for a presumed diagnosis of CMML - an intervention associated with substantial morbidity and mortality rates, which was thankfully averted due to high clinical suspicion for an alternative unifying etiology given the noted absence of dysplasia on bone marrow biopsy. Chronic myelomonocytic leukemia (CMML) is a clonal hematopoietic malignancy characterized by persistent monocytosis, cytopenias, and dysplasia [8]. The presence of a KRAS mutation in the bone marrow, coupled with monocytosis and cytopenias, initially led to a presumptive diagnosis of CMML in our patient. However, the absence of dysplasia on bone marrow biopsy and the presence of other atypical findings, such as xanthelasmas and fibrinous pleuritis, should have raised suspicion for an alternative diagnosis. This case emphasizes the importance of integrating clinical, pathological, and molecular findings to avoid misdiagnosis and ensure appropriate management.

The successful treatment of our patient with cobimetinib, a MEK inhibitor, further supports the role of Ras-MAPK pathway activation in the pathogenesis of ECD. Cobimetinib has been shown to be effective in patients with BRAF-mutated ECD [1,20]. Although this patient was BRAF-negative, he was found to have the KRAS mutation, which is further upstream within the Ras-Raf-MEK-ERK pathway. While direct KRAS inhibitors are generally ineffective, cobimetinib has been found to have promising results for this subset of patients [20]. Several studies have demonstrated that MEK inhibition can effectively suppress MAPK signaling and reduce disease burden in ECD patients with RAS mutations [1,3,5,6,20]. Our case provides further evidence for the efficacy of MEK inhibitors in RAS-mutated ECD and underscores the importance of molecular profiling to guide targeted therapy decisions.

It is important to acknowledge the limitations of this case report. First, it is a single case, and therefore, the findings may not be generalizable to all ECD patients. Second, the diagnosis of ECD was based on clinical, pathological, and molecular findings, but FDG PET/CT was not confirmatory. Finally, the follow-up period was relatively short, and the long-term efficacy and safety of cobimetinib in this patient population are limited.

Despite these limitations, this case report provides valuable insights into the diagnostic and therapeutic challenges of ECD. It highlights the importance of considering ECD in patients with unexplained multisystem involvement, even in the absence of typical clinical or radiological findings. It also emphasizes the value of comprehensive molecular profiling to identify potentially targetable mutations and guide personalized treatment strategies. Future research should focus on further elucidating the pathogenesis of ECD, developing more effective therapies, and establishing clear diagnostic and treatment guidelines for this rare and challenging disease.

## Conclusions

This case highlights the diagnostic challenges inherent in ECD, particularly when presentations deviate from classic patterns or masquerade as another disease process. The patient's initial constellation of seemingly unrelated symptoms, such as xanthelasmas, fibrinous pleuritis, arthralgias, and cytopenias, led to a delayed diagnosis and consideration of alternative diagnoses such as CMML. That alone stresses the importance of avoiding anchoring bias, particularly with such a drastically different course in management strategies. Recognition of the xanthelasma's foamy histiocytic infiltrates (CD68+/S100-), coupled with molecular profiling revealing a KRAS mutation, proved critical in redirecting the diagnostic trajectory and enabling targeted therapy with cobimetinib.

This report contributes to the growing body of literature on atypical ECD presentations, particularly those with Ras pathway mutations. It emphasizes the need to consider ECD even in the absence of typical skeletal findings and to pursue comprehensive molecular profiling in BRAF-negative cases. The patient's positive response to cobimetinib further supports the role of MEK inhibition in managing RAS-mutated ECD, although further research is needed to fully elucidate the long-term efficacy and optimal treatment strategies for this patient subgroup.

This case reinforces the importance of maintaining a broad differential diagnosis, integrating clinical, pathological, and molecular findings, and considering rare entities like ECD in patients with unexplained multisystem involvement. Early recognition and targeted therapy can significantly improve outcomes, prevent unnecessary interventions, and enhance the quality of life for individuals affected by this rare and often debilitating disease. Moving forward, continued efforts to improve diagnostic accuracy, develop novel therapeutic approaches, and establish clear clinical guidelines are essential for appropriate recognition and optimizing the care of patients with ECD.
